# ASSOCIATION BETWEEN FRACTION OF EXHALED NITRIC OXIDE AND SPIROMETRY DATA AND CLINICAL CONTROL OF ASTHMA IN CHILDREN AND ADOLESCENTS

**DOI:** 10.1590/1984-0462/;2018;36;1;00015

**Published:** 2018-01-15

**Authors:** Luanda Dias da Silva Salviano, Karla Delevedove Taglia-Ferre, Sandra Lisboa, Ana Carolina Carioca da Costa, Hisbello da Silva Campos, Maria de Fátima Pombo March

**Affiliations:** a Instituto Nacional de Saúde da Mulher, da Criança e do Adolescente Fernandes Figueira da Fundação Oswaldo Cruz (IFF/Fiocruz), Rio de Janeiro, RJ, Brasil.; b Hospital Infantil Nossa Senhora da Glória, Cachoeiro de Itapemirim, ES, Brasil.; c Universidade Federal do Rio de Janeiro, Rio de Janeiro, RJ, Brasil.

**Keywords:** Asthma, Spirometry, Children, Adolescents, Nitric oxide, Asma, Espirometria, Crianças, Adolescentes, Óxido nítrico

## Abstract

**Objective:**

To evaluate the association between fraction of exhaled nitric oxide (FeNO) values and forced expiratory volume in the first second (FEV1) and the level of asthma control, as proposed by the Global Initiative for Asthma (GINA), in asthmatic children and adolescents attended at the National Institute of Women, Children and Adolescents Health Fernandes Figueira of Fundação Oswaldo Cruz (IFF/FIOCRUZ).

**Methods:**

This was a cross-sectional study, with a review of medical records of 90 asthmatics between 7 and 17 years old, who were followed up at the IFF/FIOCRUZ Asthma Outpatient Clinic and were referred to perform respiratory function tests (RFT)between March 2013 and September 2014. After classification according to GINA, patients performed complete spirometry and FeNO measurement. Subsequently, they were separated into two groups: regular and non-regular inhaled corticosteroid (ICS) use, regardless of the ventilatory pattern in spirometry.

**Results:**

The association between FEV1 values and the degree of asthma control according to GINA (p=0.001) was observed in all patients, regardless of ICS use, but there was no association between FEV1 and levels of FeNO.

**Conclusions:**

The correlation observed between GINA and FEV1 reinforces the importance of spirometry in the clinical follow-up of these patients. Although no association was found between the value of FeNO and the degree of asthma control and FEV1, FeNO may be an early method to detect airway inflammation, even before the symptoms and spirometric changes.

## RESUMO

**Objetivo:**

Avaliar a associação entre os valores da fração exalada de óxido nítrico (FeNO) e do volume expiratório forçado no primeiro segundo (VEF_1_) e o grau de controle da asma, segundo proposta do *Global Initiative for Asthma* (GINA), em crianças e adolescentes asmáticos atendidos no Instituto Nacional de Saúde da Mulher, da Criança e do Adolescente Fernandes Figueira da Fundação Oswaldo Cruz(IFF/FIOCRUZ).

**Métodos:**

Estudo transversal, com revisão de prontuários de 90 asmáticos entre 7 e17 anos de idade, acompanhados no Ambulatório de Asma do IFF/FIOCRUZ e encaminhados ao setor de Prova de Função Respiratória (PFR) entre março de 2013 e setembro de 2014. Após classificação segundo o GINA, os pacientes realizaram espirometria completa e medida da FeNO. Posteriormente, foram separados em dois grupos: em uso regular e não regular de corticosteroide inalatório (CI), independentemente do padrão ventilatório na espirometria.

**Resultados:**

Observou-se associação entre os valores de VEF_1_ e o grau de controle da asma segundo o GINA (p=0,001) em todos os pacientes analisados, independentemente do uso de CI, mas não houve associação entre os valores de VEF_1_ e os níveis da FeNO.

**Conclusões:**

A correlação observada entre o GINA e o VEF_1_ reforça a importância da espirometria no seguimento clínico desses pacientes. Embora não tenha sido detectada associação entre o valor da FeNO e o grau de controle da asma e o VEF_1_,a FeNO pode constituir um método precoce para detectar inflamação nas vias aéreas, antes mesmo dos sintomas e das alterações espirométricas.

## INTRODUCTION

Asthma is a heterogeneous disease, usually characterized by chronic airway inflammation.^1^ It is defined by the history of respiratory symptoms such as wheezing, breathlessness, chest tightness, and coughing, which vary over time and in intensity, along with variable expiratory airflow. Asthma is a global health problem that affects approximately 300 million individuals of all ages and ethnic groups in many countries. It is a major respiratory disease in childhood which brings social and economic impacts and causes around 250,000 premature deaths annually,^2^ ^,^ ^3^ a number probably underestimated.^4^

The evaluation of the clinical control of asthma proposed by the Global Initiative for Asthma (GINA) is based on clinical and functional parameters. Clinical parameters define the disease as controlled, partly controlled, and uncontrolled,^5^ and functional parameters aim at evaluating airflow limitation and contributing to determine the most appropriate treatment.^6^

Asthma severity and response to treatment can be assessed by clinical monitoring and lung function parameters: forced expiratory volume in the first second (FEV_1_), FEV_1_/forced vital capacity (FVC) ratio, forced expiratory flow between 25 to 75% of FVC (FEF_25-75%)_, slow vital capacity (SVC), and peak expiratory flow (PEF) obtained by spirometry. However, neither clinical nor functional monitoring enables the evaluation of the intensity of patient’s airway inflammation.^7^ ^,^ ^8^ In this context, the inflammatory marker can be an important tool for monitoring asthma.

The fraction of exhaled nitric oxide (FeNO) is a noninvasive method which enables to evaluate the degree of airway inflammation. It provides immediate results and shows high values in asthmatics.^9^ ^,^ ^10^ Current evidence suggests that early detection of inflammation may direct therapy among asthmatics.^11^ As asthma is the result of various inflammatory mechanisms which may overlap or succeed, the measurement of exhaled nitric oxide can be an important therapeutic tool to guide these patients.

Thus, the aim of this study was to evaluate the association of spirometric data with levels of inflammation of the airways by means of FeNO and the degree of clinical management of children and adolescents with asthma. The hypothesis was that asthmatic children and adolescents with more severe lung function have higher FeNO levels and poorer level of clinical control, and that lung function is associated with clinical control of asthma.

## METHOD

Cross-sectional study with retrospective chart review of 90 patients diagnosed with asthma, who were aged between 7 and 17 years and were assisted at the asthma outpatient unit of the *Instituto Nacional de Saúde da Mulher, da Criança e do Adolescente Fernandes Figueira* of *Fundação Oswaldo Cruz* (IFF/FIOCRUZ) and referred to the Pulmonary Function Testing (PFT) unit of the institution from March 2013 to September 2014. This study was approved by the Research Ethics Committee of the *Universidade Federal Fluminense* (CEP / UFF) - University Hospital Antônio Pedro of the School of Medicine of UFF - HUFMUFF-CAAE: 47567715.1.0000.5243.

All adolescents and children assisted during the study period were assessed according to the GINA 2016 classification, regardless of the clinical level of control and severity.^1^ First, the level of asthma control was defined by the doctor according to the recommendation of GINA. Thereafter, patients underwent measurement of FeNO and full spirometry. In addition to FeNO values and spirometric parameters (FEV_1,_ FVC, FEV_1_/FVC, FEF_25-75%_), descriptions of anthropometric characteristics (age, sex and body mass index - BMI) and regular dose of inhaled corticosteroids - IC (budesonide or equivalent), which is known as everyday use for at least three months, were collected from the patient´s charts. This period is the minimum time for the symptomatic asthma control.^6^

During medical examination, adhesion to medication was assessed by clinical judgment and reports from the patient and family. In this medical consultation, IC dose was also adjusted according to clinical and functional findings. The criteria established by GINA have been used to categorize the IC:^1^ children aged 6-11 years: 100-200 mcg = low dose, 200-400 mcg = moderate dose, and >400 mcg = high dose; children aged >12 years: 200-400 mcg = low dose, 400-800 mcg = moderate dose, and >800 = high dose. Subsequently, patients were divided into two groups: those who were using IC and those who were not, regardless of the results of spirometry. Children and adolescents with associated pulmonary comorbidity, as well as those who did not properly perform spirometric tests and FeNO were excluded from the study.

The level of asthma control was assessed according to GINA 2016. Therefore, for the classification of severity, patients responded to the questionnaire which assesses control of asthma established over the past four weeks. The following parameters were analyzed: symptoms, nocturnal awakenings, use of rescue medication, activity limitation, and FEV_1_ value. Finally, patients were classified as controlled, partly controlled, and uncontrolled asthma. PEF is also a functional parameter that can be used in this classification; however, it is not routinely performed in all children and adolescents in the Asthma Clinic of IFF. For this reason, PEF was not included in this study.

FeNO measurement was performed according to the 2011 recommendations of the *American Thoracic Society* (ATS)^12^ and prior to spirometry, to do not modify its values. A portable analyzer Niox^®^ Mino (Aerocrine, Sweden), in which the expiratory flow is maintained at 50 mL/s and is controlled by acoustic emission signal was used. The values of FeNO were stratified into: low (<20 ppb for children under 12 years of age and <25 ppb for those children older than 12 years), intermediate levels (20-35 ppb for children under 12 years of age and 35-50 ppb for children above 12 years of age), and high levels (>35 ppb for children under 12 years of age and >50 ppb for those older than 12 years).

All spirometry analyzed in this study were conducted according to the standards set by the *American Thoracic Society and European Respiratory Society* (ATS/ERS), 2005.^13^ The examinations were performed by the same professional, and all expiratory maneuvers were filed in their original sequence. The spirometer used was a Jaeger, Master Scope^®^ (Viasys Healthcare, Hoechberg, Germany). The examinations were performed with and without bronchodilator. Patients met all the requirements for the exam, such as do not use bronchodilator substance at least 4 hours before the test, hold the test for 2 weeks after respiratory infection, and delay the examination for 7 days after hemoptysis and suspension of bronchodilators of short (6 hours) or long (12 hours) duration before the test. For bronchodilator response, 400 micrograms of albuterol were inhaled through the spacer device. Fifteen minutes after administration of the drug, each child underwent new spirometry to evaluate the bronchodilator response. Spirometry was considered normal when FEV_1_/FVC showed a value above the upper limit of normal (ULN) of the 5th percentile. The respiratory disorder, when observed, was characterized as obstructive when FEV_1_/FVC ratio was below the lower limit of normal (LLN) of the 5th percentile. Disorder severity level was considered mild, when FEV_1_ was at ≥70%; moderate, when it reached between 60-69%; moderate severe between 50-59%; severe between 35-49%; and life threatening, when <35%.

In the statistical analysis, descriptive measures appropriate to the various variables were used, including frequencies and percentages (categorical variables), means and standard deviations (continuous variables with normal distribution), and median, minimum and maximum (continuous variables with nonnormal distribution). The assumption of normality was verified by the Shapiro-Wilk test. The analysis of variance (ANOVA) was applied to verify if the FEV_1_ values differed according to FeNO levels in 90 asthmatics and in those patients classified according to the use or not of IC and to assess if the FEV_1_ vary significantly in the different ranges of asthma control classification among those who regularly used IC. In the evaluation of that relationship among patients who were not using ICs, the nonparametric Kruskal-Wallis test was applied. Regarding clinical and functional characteristics as well as IC dose, the chi-square test was used to check for association with levels of FeNO. Fisher exact test was applied when we observed at least one expected frequency lower than five. The statistical analyzes were performed using IBM Corp. Released 2013. IBM SPSS Statistics for Windows, Version 22.0. Armonk, NY: IBM Corp., based on a 5% significance level.

## RESULTS

Of the 90 asthmatic adolescents and children evaluated, 68 were treated with IC and 22 were not. Average age was 11.5±2.9 years, with a predominance of males (66.7%). The median levels of FeNO was 35.5 ppb in the total population of asthmatics studied. Among the 90 children and adolescents (61.1%) evaluated, 55 had obstructive pattern. Table 1 shows the general characteristics of the studied population.

**Table 1:** General Characteristics of the study population (n=90).

Characteristic	n=90
Age (years)	11.5 (± 3.0)
Sex	
Male	60 (66.7%)
Female	30 (33.3%)
Weight (kg)	43.2 [16.0 to 118.3]
Height (cm)	147.7 [114.0 to 185.0]
BMI	20.5 [12.3 to 40.9]
FeNO (ppb)	35.5 [7.0 to 134.0]
Low	25 (27.8%)
Intermediate	31 (34.4%)
High	34 (37.8%)
Pulmonary function test	
FVC (ml)	2.80 [0.7 - 5.3]
FVC (%)	100.00 [44.0 - 130.0]
FEV_1_ (ml)	2.26 [0.6 - 4.7]
FEV_1_ (%)	92.20 [0.4 - 132.0]
FEV_1_/ FVC	80.10 (±52.0)
Pulmonary pattern	
Nonobstructive	35 (38.9%)
Obstructive	55 (61.1%)
Level of asthma control according to GINA	
Controlled	45 (50.0%)
Partly controlled	31 (34.4%)
Uncontrolled	14 (15.6%)
Inhaled corticosteroid use	
No	22 (24.4%)
Yes	68 (75.6%)
Inhaled corticosteroid dose (n=68)	
Low	27 (39.7%)
Moderate	37 (54.4%)
Upper	4 (5.9%)

Values expressed as n (%), mean (± standard deviation) or median [minimum - maximum]. BMI: body mass index; FeNO: fraction of exhaled nitric oxide; ppb: parts per billion; FVC: forced vital capacity; FEV_1_: forced expiratory volume in one second; GINA: *Global Initiative for Asthma.*

**Figure 1:**
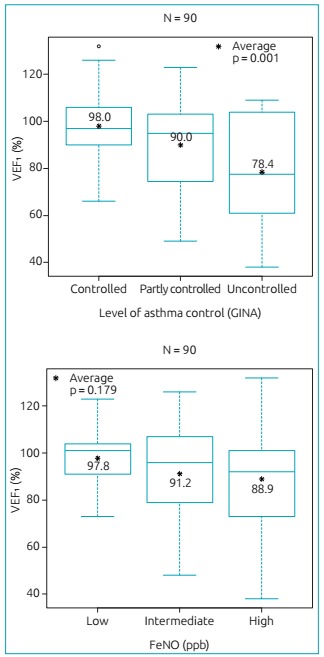
Distribution of values of forced expiratory volume in one second_,_ according to asthma control level of the *Global Initiative for Asthma* and fraction of exhaled nitric oxide levels, in parts per billion, for the study population (n=90).

In Figure 1, one can observe a significant association of FEV_1_ with GINA *(*p=0.001) in this population. A correlation was found between the classification of asthma control, according to GINA, and FEV_1_ values. However, there was no significant association of FEV_1_ with FeNO *(*p=0.179).

**Figure 2:**
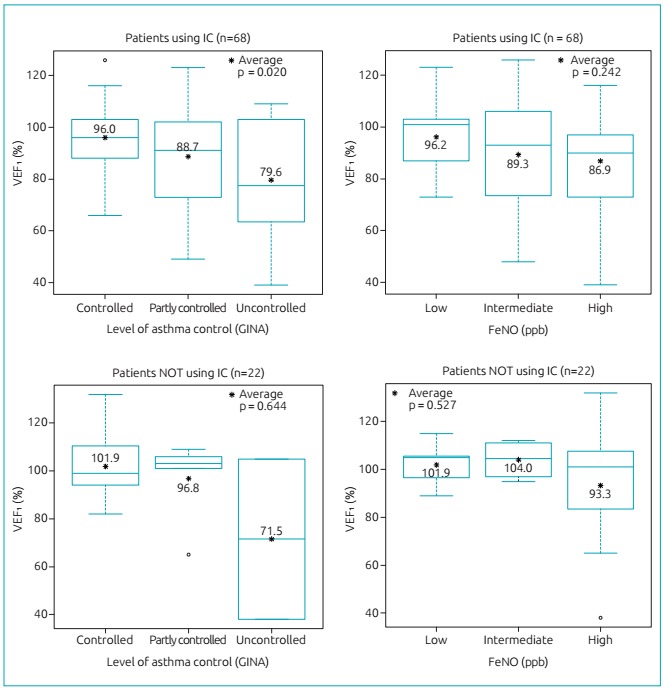
Distribution of values of forced expiratory volume in one second, according to the degree of asthma control of the *Global Initiative for Asthma* and the levels of fraction of exhaled nitric oxide, in parts per billion, for the study population, differentiated by the use of inhaled corticosteroids.

In patients using IC, FEV_1_ percentages gradually decreased as the degree of asthma control has deteriorated *(*p=0.020). However, this association was not significant between FEV1 and FeNO *(*p=0.242) for asthmatics using IC. When the analysis was performed on those not using IC, it was evident that there was no association between both FEV_1_ and GINA*(*p=0.644) and FEV_1_ and FeNO *(*p=0.527) (Figure 2).

In Table 2 it can be observed that, among patients treated with IC and exhaled levels above 20 ppb, 66.7% had controlled asthma; 72.0% normal pulmonary function; and 81.4% used low dose of IC. Although the findings did not show statistical difference, exhaled nitric oxide levels were high even when the asthma was controlled and lung function was normal.

**Table 2:** Clinical and functional characteristics and inhaled corticosteroids dose in relation to the levels of fraction of exhaled nitric oxide among the treated population (n=68).

	FeNO			p-value
	Low n (%)	Intermediate n (%)	High n (%)	
GINA				
Controlled	10 (33.3)	9 (30.0)	11 (36.7)	0.57
Partly controlled	6 (23.1)	11 (42.3)	9 (34.6)	
Uncontrolled	2 (16.7)	7 (58.3)	3 (25.0)	
Pulmonary pattern				
Obstructive	11 (25.6)	17 (39.5)	15 (34.9)	1.00
Nonobstructive	7 (28.0)	10 (40.0)	8 (32.0)	
IC dose				
Low	5 (18.5)	13 (48.1)	9 (33.3)	0.67
Moderate	12 (32.4)	12 (32.4)	13 (35.1)	
High	1 (25.0)	2 (50.0)	1 (25.0)	

FeNO: fraction of exhaled nitric oxide; GINA: *Global Initiative for Asthma*; CI: inhaled corticosteroids.

## DISCUSSION

This study examined the association between values of the FeNO levels and baseline FEV_1_ and the level of asthma control according to GINA in a sample of 90 asthmatic children and adolescents who were using IC or not.

There was association of baseline FEV_1_ with the degree of asthma control according to GINA classification, regardless of the use of IC, since FEV_1_ percentages decreased gradually as the degree of asthma control has deteriorated (p=0.001). Fuhlbrigge *et al.* in 2006 found similar results,^14^ which concluded that FEV_1_ is widely associated with important clinical outcomes in children with asthma such as symptoms, exacerbations, and use of health care. Rather, Vidal *et al.* ^15^ in study with 88 patients between 12 and 17 years of age found that FEV_1_ is not an adequate instrument to monitor asthma control in children and adolescents, as much of asthmatic children and adolescents have FEV_1_ values within the range of normality.

Measurement of FeNO is among the biomarkers currently studied to assess airway inflammation. Although there are still uncertainties in the interpretation of results, its value has been correlated with the proportion of eosinophils in induced sputum and with bronchial hyperreactivity.^7^ Studies conducted by Verini *et al.* ^16^ and Jongste *et al.* ^17^ suggested that the measurement of FeNO is a valid and effective inflammatory marker in monitoring the treatment of asthma in pediatric patients. Among the positive aspects of the method are the noninvasive nature and easiness to perform. However, high costs still hinder its wide use in public health services. In 2011, the ATS^12^ established FeNO cutoff points in the detection of eosinophilic inflammation. For children under 12 years of age, values exceeding 35 ppb evidence eosinophilic inflammation.^18^ In our study, regardless of the degree of asthma control (GINA), patients showed median greater than 35.5 ppb.

In this study, there was no association of spirometry with FeNO levels, regardless of IC use. Despite the absence of statistical significance, it was possible to observe that FEV_1_ gradually decreased as the FeNO level increased. These results are similar to the study of Paro-Hector *et al.,* ^19^ which was conducted with 26 asthmatic children and adolescents aged 6 to 18 years. However, Colon-Semidey *et al.,* ^20^ in a study with 49 children and adolescents aged 5 to 16 years, found significant correlation between the reversibility of airway obstruction and FeNO values, although they also did not detect statistical significance between FeNO and the values of FEV_1_ before bronchodilator use. In another study with 230 atopic children, a statistical correlation between FeNO and FEV_1_ was found only in clinically controlled patients treated with doses of beclomethasone greater than 200 mcg/day or equivalent, which enabled to conclude that the relationship between airflow limitation, bronchodilator response, and exhaled nitric oxide is critically dependent on the use and dosage of IC.^21^

According to the classification proposed by GINA, 36.7% of patients using IC were classified as controlled and had high levels of FeNO (>35 ppb). In 2/68 (16.7%) of children and adolescents studied, asthma was uncontrolled and FeNO levels were low (<2 ppb). In contrast to our study, Alvarez-Gutierrez *et al.* found greater proportion of patients with uncontrolled asthma and low levels of FeNO (40%) than with controlled asthma and high levels of FeNO (26%).^22^ Results similar to those of this study have been described by Khalili *et al.,* which compared the FeNO levels with five forms of clinical assessment of asthma, among them the GINA. In that study, 38% of controlled patients had high levels of FeNO (>35 ppb).^23^ According to the ATS, asthmatics with absence of respiratory symptoms and high levels of FeNO have increased risk of exacerbation.^12^

The main purpose of the classification of asthma severity is to determine the dose of drug which is sufficient to obtain patient´s disease control as soon as possible.^6^ In this study, we observed that 33.3% of patients treated with IC had controlled asthma and low levels of FeNO, regardless of IC dose. However, a limitation of the study was the analysis of association between asthma control by means of FeNO and treatment. Another study is necessary for this analysis performing serial measurements of FeNO pre- and post-treatment to evaluate the response to inhaled corticosteroid.

Among 18 patients (72%) treated with CI who had normal spirometric parameters, we found FeNO level of ≥20 ppb, regardless of the clinical control, similar to that observed by Jentzsch *et al.*,^24^ who analyzed 45 children and adolescents with persistent asthma, and by Kovesi *et al.* ^25^ who have studied 1,135 students. Both studies found high levels of FeNO in patients with clinical and spirometric normality, which demonstrates that the normal respiratory function does not reflect the absence of inflammation in the airway and that, even with normal spirometric values, it is not possible to affirm that a patient has asthma adequately controlled.^24^ Although GINA’s recommendations do not include the measurement of FeNO to decide the initiation of treatment with IC in patients with nonspecific symptoms, values greater than 50 ppb are associated with favorable response to the use of IC.^1^

The results of this study suggest the importance of spirometry in the clinical follow-up of these patients and that the measurement of FeNO can be an early method for detecting airways inflammation, even before symptoms and spirometric alterations. It is possible that this measure is also a response marker to inhaled corticosteroid therapy in children and adolescents with asthma, regardless of respiratory functional evaluation.

Apparently, the classification of clinical control of asthma proposed by GINA, the measurement of FeNO, and values of FEV_1_ are independent of each other, for analyzing different aspects of the same disease. The use of such methods associated would enable a broader and more objective assessment of asthma patients.
